# A Case of Type 2 Diabetes Mellitus with Lung Cancer Suffered from Euglycemic Diabetic Ketosis Accompanied by Adrenal Insufficiency after Immune Checkpoint Inhibitors

**DOI:** 10.1155/2024/9982174

**Published:** 2024-02-20

**Authors:** Saeko Shibasaki, Chisei Noda, Akihisa Imagawa, Sadaki Sakane

**Affiliations:** ^1^Department of Internal Medicine (Diabetes and Endocrinology), Hirakata City Hospital, 2-14-1 Kinyahon-machi, Postal Code: 573-1013, Hirakata, Osaka, Japan; ^2^Department of Internal Medicine (I), Osaka Medical and Pharmaceutical University, 2-7 Daigaku-machi, Postal Code: 569-8686, Takatsuki, Osaka, Japan

## Abstract

A 74-year-old patient with type 2 diabetes mellitus received basal-bolus insulin, insulin secretagogues, and sodium glucose transporter 2 (SGLT2) inhibitors. After immune checkpoint inhibitor treatment for lung cancer, he suffered from depressed consciousness with a urinary ketone body (3+). When all hypoglycemic treatments were discontinued, his serum blood glucose remained at 121 mg/dL. He was diagnosed with euglycemic diabetic ketosis. Endocrine loading tests revealed isolated adrenocorticotropic hormone (ACTH) deficiency as an immune-related adverse event. It was suggested that euglycemic diabetic ketosis was induced by the self-suspension of insulin and insulin secretagogues, adrenal insufficiency, SGLT2 inhibitors, and carbohydrate intake shortage.

## 1. Introduction

Immune checkpoint inhibitors (ICIs) of monoclonal antibodies against programmed cell death (PD-1), its ligand (PD-L1), or cytotoxic T lymphocyte antigen-4 (CTLA-4) have been approved as anticancer drugs. They are widely used as effective treatments for several advanced malignant diseases, including nonsmall cell lung cancer (NSCLC) [1]. However, ICIs may cause immune-related adverse events (irAEs), including hypopituitarism, primary adrenal insufficiency, thyroid dysfunction, hypoparathyroidism, and type 1 diabetes mellitus. The frequency of endocrine irAEs increases with combination therapy with anti-PD-1 and anti-CTLA-4 antibodies [2].

Sodium glucose transporter 2 inhibitors (SGLT2 inhibitors) are a new type of glucose-lowering drug. They can reduce blood glucose by inhibiting its reabsorption in proximal tubules and by promoting urinary glucose excretion [3]. SGLT2 inhibitors are widely used in the clinical treatment of type 2 diabetes mellitus (T2DM). However, SGLT2 inhibitors may also cause euglycemic diabetic ketosis. It is a life-threatening emergency and is characterized by milder degrees of hyperglycemia with blood glucose levels of <200 mg/dL with severe metabolic acidosis [4]. Euglycemic diabetic ketoacidosis is also induced by other factors, such as surgery, trauma, pregnancy, decreased caloric intake, heavy alcohol use, insulin use prior to hospital admission, or liver cirrhosis [4, 5]. However, there are no reports of euglycemic diabetic ketosis in T2DM associated with isolated ACTH deficiency induced by irAEs. In this case report, we describe the clinical features of irAE-related euglycemic diabetic ketosis in T2DM.

## 2. Case Presentation

A 74-year-old man suffered from T2DM at 58 years of age. He was treated with basal-bolus insulin at a total daily dose of 0.58 units/kg/day, canagliflozin 100 mg/day, glimepiride 1 mg/day, metformin 1000 mg/day, and dulaglutide 0.75 mg/week. His glucose control was poor, and his HbA1c level was 8.0–9.0%. He had a carbohydrate-rich diet. He also suffered from fatty liver. His body weight was 79 kg, and his body mass index was 25.8 kg/m^2^. His fasting serum C-peptide level was 0.76–1.57 ng/mL, and his postprandial C-peptide level was 2.25–6.28 ng/mL, indicating a normal to mild decrease in insulin secretion.

An abnormal lung mass was detected in regular medical checks provided by his company, and he was diagnosed with squamous cell carcinoma of the left lower lobe (clinical TNM stage T3N3M0 and NSCLC stage IIIC). He was treated with carboplatin, paclitaxel, nivolumab (anti-PD-1 antibody), and ipilimumab (anti-CTLA-4 antibody). Nivolumab and ipilimumab were regularly administered for maintenance treatment.

On the 8^th^ day of the 3^rd^ cycle of nivolumab and ipilimumab, he felt general fatigue, excessive daytime sleepiness, and appetite loss. On the 13^th^ day, those symptoms further worsened, and he discontinued injecting insulin and dulaglutide and taking all oral hypoglycemic agents. On the 19^th^ day, he was transported to the emergency room of our hospital with a high fever of 38.3°C and a depressed level of consciousness, as assessed by the Glasgow Coma Scale (E4V3M5).

On admission, his blood pressure, pulse, and respiratory rate were 118/79 mmHg, 130/min, and 24/min, respectively. His percutaneous oxygen saturation was 97% on room air. Laboratory blood and urine tests revealed the following: serum glucose, 121 mg/dL; HbA1c, 9.3%; acetoacetic acid, 2076 *μ*mol/L; *β*-hydroxybutyric acid, 4995 *μ*mol/L; urinary ketone body (3+); venous blood gas with a pH of 7.40; and C-reactive protein, 16.6 mg/dL (Table 1). Noncontrast-enhanced computed tomography showed no signs of infection in the organs of his body. Based on those findings, euglycemic diabetic ketosis was diagnosed.

**Figure 1:**
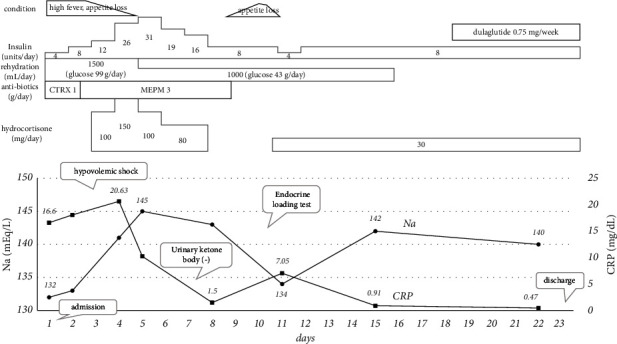
The clinical course after admission. CTRX, ceftriaxone sodium hydrate; MEPM, meropenem hydrate.

He received intravenous rehydration containing glucose, insulin, and antibiotic treatment, which are standard treatments for euglycemic diabetic ketosis and bacterial infection. However, severe unconsciousness and hypovolemic shock appeared on the 3^rd^ day of hospitalization. All 3 sets of blood and urine culture tests were negative. Therefore, we suspected that euglycemic diabetic ketosis was accompanied by adrenal insufficiency. We started 5 consecutive days of intravenous hydrocortisone supplementation (80–150 mg/day) followed by twice-daily oral hydrocortisone supplementation (30 mg/day). His condition drastically improved, and his urinary ketone body concentration became negative on the 7^th^ day of hospitalization. A rapid ACTH test was performed on the 8^th^ day to confirm the diagnosis of adrenal insufficiency (Table 1). Subsequently, a corticotropin-releasing hormone loading test was performed on the 10^th^ day and revealed secondary adrenal insufficiency. The levels of the other anterior pituitary hormones were normal (Table 1). Enhancement and dynamic magnetic resonance imaging of the pituitary showed no enlargement of the pituitary gland or stalk, mass formation, or inflammation. We established a diagnosis of isolated ACTH deficiency. We judged it to be an irAE and considered that euglycemic diabetic ketosis was secondarily provoked. According to the Common Terminology Criteria for Adverse Events v5.0 (CTCAEv5.0) [6], his irAE grade was evaluated as level 4, most severe or life-threatening. He lost 6.2 kg of body weight in 2 months. He was discharged on the 23^rd^ day while receiving hydrocortisone supplementation. His diabetes was well-controlled by basal-bolus insulin and weekly glucagon-like peptide-1 receptor agonist injection therapy (Figure 1).

## 3. Discussion

We reported the case of a patient with T2DM and NSCLC who suffered from euglycemic diabetic ketosis and adrenal insufficiency caused by isolated ACTH deficiency as an irAE. This is the first reported case in which euglycemic diabetic ketosis associated with adrenal insufficiency was recognized in a T2DM patient with residual insulin secretion. To date, three reports have recognized diabetic ketoacidosis with isolated ACTH deficiency as an irAE [7–9]. All patients had type 1 diabetes mellitus accompanied by isolated ACTH deficiency. Namely, in these patients, diabetic ketoacidosis developed in a background of absolute insulin deficiency. However, in our patient, euglycemic diabetic ketosis developed in a patient with a preserved insulin secretion capacity.

**Figure 2:**
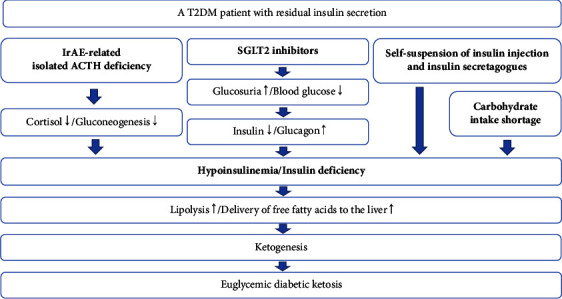
Diagram of the development of euglycemic diabetic ketosis.

Several clinical factors were suggested to induce euglycemic diabetic ketosis in T2DM patients with residual insulin secretion. It might be true that irAE-related isolated ACTH deficiency created an opportunity to develop euglycemic diabetic ketosis. However, that alone was not sufficient for the development of diabetic ketosis because it could not cause insulin deficiency [10]. In addition to irAE-related isolated ACTH deficiency, self-suspension of insulin injection and insulin secretagogues, SGLT2 inhibitors, and carbohydrate intake shortage might have contributed to the development of euglycemic diabetic ketosis. Next, we describe the mechanism that is presumed to underlie the development of euglycemic diabetic ketosis in our patient in detail (Figure 2).

The self-suspension of insulin injection and insulin secretagogues may have accelerated insulin deficiency. The patient required a large dose of insulin plus insulin secretagogues to control his blood glucose as usual. During sick days, the demand for insulin increases due to decreased insulin sensitivity [11]. Six days of consecutive self-suspension would certainly promote insulin deficiency.

Adrenal insufficiency, SGLT2 inhibitors, and carbohydrate intake shortage might keep blood glucose levels lower. Cortisol plays a role in stimulating gluconeogenesis and inhibiting glycogenesis to prevent hypoglycemia [12, 13]. Thus, adrenal insufficiency leads to hypoglycemia [12]. SGLT2 inhibitors lower blood glucose levels by inhibiting the reabsorption of glucose in proximal tubules and by promoting urinary glucose excretion [3]. The half-life of canagliflozin in T2DM repeatedly administered at 100 mg/day is 11.8 ± 3.2 hours [14]. However, the prolonged influence of SGLT2 inhibitors and delayed recovery from euglycemic diabetic ketoacidosis have been reported, even at 6–10 days [15, 16]. Uenishi et al. reported that dehydration and a decreased renal function were one of the reasons for the prolonged influence of SGLT2 inhibitors [15], as was also shown in our patient. Carbohydrates are the main source of glucose, and it is well known that a carbohydrate-restricted diet reduces blood glucose levels [17]. Our patient had a medical record of fasting serum C-peptide level of 0.76 ng/mL at 102 mg/dL of blood glucose. So, insulin secretion had been inhibited during adrenal insufficiency.

Taken together, at first, self-suspension of insulin injection and insulin secretagogues led to relative insulin deficiency, resulting in the increased production of ketone bodies. Second, adrenal insufficiency, SGLT2 inhibitors, and carbohydrate intake shortage kept blood glucose levels lower despite insulin deficiency. Finally, pancreatic *β* cells cannot compensate for decreased insulin secretion, resulting in temporary severe insulin deficiency. Temporary severe insulin deficiency promotes lipolysis, the delivery of free fatty acids to the liver, ketogenesis, and ultimately, euglycemic diabetic ketosis [4, 5] (Figure 2).

In addition to temporary severe insulin deficiency, the upregulation of glucagon secretion contributes to the overproduction of ketone bodies although serum glucagon levels were not measured. The administration of SGLT2 inhibitors stimulates the secretion of glucagon, which is mediated by a decrease in insulin secretion [5] or a direct effect of SGLT2 inhibitors on pancreatic *α*‐cells [18].

Our patient showed diabetic ketosis due to hypoinsulinemia but not diabetic ketoacidosis. McNulty et al. [19] reported a case of newly diagnosed type 1 diabetes mellitus with Addison's disease, which showed only diabetic ketosis and did not progress to diabetic ketoacidosis. Sheung et al. [20] reported that the degree of acidosis was very mild in diabetic ketoacidosis caused by self-discontinuation of insulin injection in type 1 diabetes mellitus with Addison's disease. It was speculated that ketone body synthesis due to insulin deficiency were partly offset by adrenal insufficiency [19, 20].

In conclusion, we reported the case of a patient with T2DM who was treated with ICIs for NSCLC and developed euglycemic diabetic ketosis due to several clinical factors. It is necessary for healthcare professionals to recognize that such patients have a high risk of developing euglycemic diabetic ketosis.

## Figures and Tables

**Table 1:** Patient laboratory data from the time of administration and the results of endocrine loading tests.

*Complete blood count*			*Biochemical blood test*		
WBC	9640	/*μ*L	AST	26	U/L
Neut	64.2	%	ALT	11	U/L
Lymp	18.6	%	LDH	168	U/L
Mono	6.7	%	*γ*-GTP	15	U/L
Eos	9.9	%	CPK	464	U/L
Baso	0.6	%	TP	6.0	g/dL
RBC	4.96 × 10^6^	/*μ*L	ALB	2.8	g/dL
Hb	14.3	g/dL	BUN	26.3	mg/dL
Ht	44.2	%	Cr	1.07	mg/dL
PLT	463 × 10^3^	/*μ*L	Na	132	mEq/L
*Urinary test*			K	5.1	mEq/L
Specific gravity	1.030		Cl	97	mEq/L
pH	6.0		Modified Ca	11.6	mg/dL
Sugar	(4+)		P	3.8	mg/dL
Blood	(−)		CRP	16.6	mg/dL
Protein	(−)		NH3	21	*μ*g/dL
Ketone body	(3+)		T-Bil	0.52	mg/dL
WBC	(−)		AMY	32	U/L
Bacteria	(−)		Vit B12	57.2	ng/mL
*Venous blood gas*			Cortisol	7.1	*μ*g/dL
pH	7.40		CYFRA	3.0	ng/mL
PCO2	28.7	torr	eGFR	53	ml/min/L
PO2	77.0	torr	*Diabetes/thyroid blood test*		
HCO3	17.4	mmol/L	Glucose	121	mg/dL
SBE	−6.5	mmol/L	HbA1c	9.3	%
Lac	7.0	mg/dL	C-peptide	1.47	ng/mL
*Venous ketone body*			freeT4	1.21	ng/dL
Total ketone body	7071	*μ*mol/L	TSH	2.850	*μ*IU/mL
Acetoacetic acid	2076	*μ*mol/L	GADA	<5.0	U/mL
3-hydroxybutyric acid	4995	*μ*mol/L	Tg-Ab	24.9	U/mL
			TPO-Ab	3.0	U/mL
*Endocrine loading tests*					
*Rapid-ACTH test*					
	0 min	30 min	60 min		Unit
ACTH	<1.5				pg/mL
Cortisol	0.2	5.0	6.6		*μ*g/dL
*CRH, TRH and GnRH loading tests*					
	0 min	30 min	60 min	90 min	Unit
ATCH	<1.5	4.7	2.0		pg/mL
Cortisol	0.5		1.2	0.9	*μ*g/dL
PRL	33.3	93.1	86.7		ng/mL
TSH	2.34	9.54	9.01		*μ*IU/mL
LH	6.6	33.0	38.0		mIU/mL
FSH	15.6		24.6	27.3	mIU/mL
*GHRP2 loading test*					
	0 min	15 min	30 min	45 min	60 min
GH	0.88	82.6	96.4	78.7	43.9
IGF-1	122	(Units of GH, IGF-1; ng/mL)			

CYFRA, cytokeratin 19 fragment; GADA, anti-glutamic acid decarboxylase antibody; Tg-Ab, anti-thyroglobulin antibody; TPO-Ab, anti-thyroid peroxidase antibody; ACTH, adrenocorticotropic hormone; PRL, prolactin; TSH, thyroid stimulating hormone; LH, luteinizing hormone; FSH, follicle stimulating hormone; GH, growth hormone; IGF-1, insulin-like growth factor-1; CRH, corticotropin-releasing hormone; TRH, thyrotropin releasing hormone; GnRH, gonadotropin releasing hormone; GHRP2 growth hormone releasing peptide 2.

## Data Availability

The data used to support the findings of this study are available from the corresponding author upon reasonable request.
